# What Geohistory Can Teach Us About Fundamental Causes of Health Inequities

**DOI:** 10.1089/heq.2021.0191

**Published:** 2022-09-08

**Authors:** Timothy R. Rebbeck

**Affiliations:** Division of Population Science, Department of Medical Oncology, Dana-Farber Cancer Institute and Zhu Family Center for Global Cancer Prevention and Department of Epidemiology, Harvard TH Chan School of Public Health, Boston, Massachusetts, USA.

**Keywords:** geospatial, inequity, intersectionality

## Abstract

The causes of cancer health inequities are complex, multilevel, and intersectional. The typical disciplines and data used to address these inequities focus on public health, health services, clinical, and fundamental science. Fundamental causes such as systemic racism are a source of much health inequity, but a broader scope of fundamental causes may be considered. Geohistorical events may intersect with other fundamental causes of health inequities. In this study, an example of relationships between ancient geological events, slavery, and subsequent effects of systematic racism are identified. These relationships support the hypothesis that health inequities have deep and complex origins. Geohistorical factors precede social, economic, and political influences on health inequities, and suggest that a full understanding of cancer health inequities and their elimination may be informed by geohistorical events. Thus, addressing inequities may involve disciplines not typically involved in health equity collaborations, including geography, history, economics, political science, and others.

Numerous studies examining the causes of health inequities focus on the current landscape of multilevel influences that include fundamental causes such as systemic racism, segregation and discrimination; proximal factors such as genetics, biology, individual risk factors, and individual demographics; intermediate factors including physical and geospatial context, social relationships, and social context; and distal fundamental causes including institutional context and social conditions and policies.^1^ These factors do not result in inequities in health in isolation but manifest their effects through complex intersectional relationships as measured by race and socioeconomic position.^2^ Importantly, intersectionality of factors that lead to health inequities are not static but evolve with changing political, economic, policy, and social circumstances. Some of these factors may have their origin well beyond data that we consider in usual health equity frameworks.

Health inequities are, therefore, an evolving consequence of historical sociopolitical events that in the United States have culminated in the systemically and structurally racist society we observe today. The health inequities literature considers historical context that has led to the current inequity in health across race, ethnicity, gender, residence, and other groupings. The literature tends to focus on relatively recent events, including the historical transatlantic slave trade and subsequent manifestations of systematic racism in the United States. However, the origins of the sociopolitical and economic drivers of health inequities may include more complex roots than recognized by events of the past 400 years.

A number of authors have identified a relationship of ancient geological events on current social issues such as voting patterns.^3^ This fascinating observation is not limited to political phenomena but may link to determinants of health as well. In the late Cretaceous period (65–115 million years ago), the Coniacian Epeiric Sea divided what is now the United States into two land masses: Laramidia to the West and Appalachia to the East^4^ (Fig. 1A). The southern coast of Appalachia comprised a band of fossiliferous sedimentary geological formations that are visible in current geological maps^5^ (Fig. 1B). This geological band became used in the past few centuries for farming because of its fertile soil.

**FIG. 1.**
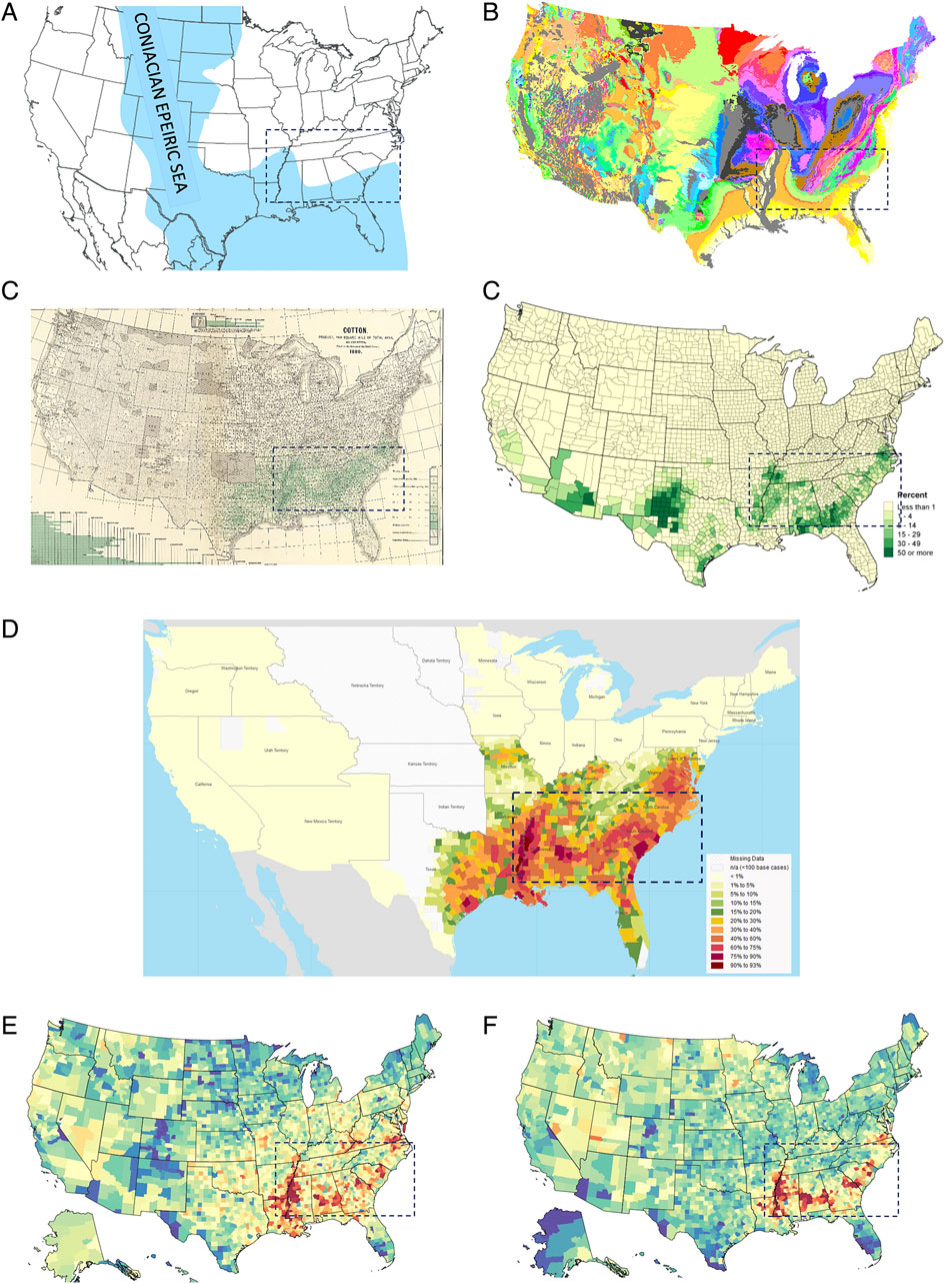
Ancient influences on current cancer inequities. **(A)** The southern edge of Appalachia along the Coniacian Epeiric Sea in the Cretaceous period.^21^ **(B)** The effects of the Cretaceous sea-land border in current geological features of the Southern United States, indicated by the *yellow arc* in MS, AL, and GA. **(C)** Cotton production in 1880 and 2007.^22^ **(D)** Distribution of the enslaved population of the United States, 1860.^23^ **(E)** County-level mortality from breast cancer, 2014.^9^ **(F)** County-level mortality from prostate cancer, 2014.^9^ The *blue dash-outlined box* represents the area of primary interest to this presentation.

In particular, cotton farming^6^ (Fig. 1C) was highly successful in this region. Along with cotton farming came the enslavement of large numbers of Africans and their descendants. Indeed, this region had among the highest numbers of enslaved individuals before the civil war, and continues to have a high percentage of African American residents^7^ (Fig. 1D). With slavery and subsequent persistent systemic racism came a wide range of social influences, including policies that denied access to quality education, health care, housing, employment, and other social and economic opportunities. The legacy of slavery continues to affect health and health inequities.

Today, the geological formation that arose during the Cretaceous period is often referred to as the “Black Belt.”^8^ Formerly called the “cotton belt,” this region was renamed for the fertile black soil that is characteristic of the region. The Black Belt includes a high proportion of African Americans, many of whom continue to live under social conditions that are not consistent with equity in health compared with other regions of the country or other racial and ethnic groups. These include high rates of poverty, lower literacy, limited transportation, and limited access to health care. As shown in Figure 1E, inequities are clearly visible through high rates of both breast and prostate cancer mortality.

Even if today's health inequities colocate with geological spaces established over 65 million years ago,^9^ do these observations have any relevance to our understanding or amelioration of health inequities? The example presented earlier offers an opportunity to consider the complex intersectional nature of health inequities that have a historical and geospatial component. Ancient geology produced conditions that were agriculturally and economically favorable for slavery to take hold, leading directly to structural racism, and in turn impacting on the health status of those living in that region to this day. Those events determined that this region would remain a rural largely agricultural economy, without opportunity to develop a diverse economy or support advanced infrastructure, education, and health systems.

These patterns can be seen in any number of maps identifying social conditions in the Black Belt and other areas. Geospatial observations can thus play a useful role in identifying communities of need, areas in which services or resources are lacking, where risk factor distributions are unfavorable, and where disease rates are high. These geographic areas of need may lie outside of the political boundaries typically used to capture data or describe cancer rates. By visualizing the consequences of political and economic history on current maps, patterns of health inequity may be identified that can guide resources, policies, and interventions to those neighborhoods, counties, or census tracts where populations in the greatest need live.

It is not necessary to go back 65 million years to find geohistorical patterns that are causative of or correlated with health inequities, and to use this knowledge to identify solutions. There are many examples in the literature of neighborhood and contextual features that have direct public health relevance. Simple maps can provide information in this regard, but sophisticated analytical tools are available that thoughtfully characterize geospatial patterns when link with population disease data.^10^ Patterns of exposure to greenness, noise, pollution, crime, contextual socioeconomic factors, transportation, and many other historically determined influences on a community's collective exposure can be identified,^11–14^ and interventions and policy decisions that ameliorate these exposures can be developed.^15–17^ Importantly, geospatial data can inform community-level interventions that efficiently focus resources to those areas of greatest need.

A concern with the incorporation of geospatial and geohistorical data in the discussion of health disparities is that of geographic determinism, which was raised as early as 1817 by Karl Ritter in his treatise die Erdkunde: the concept that human behavior (and thereby health) is a consequence of the physical environment. Although geographic determinism is a general concern in this field, the purpose of this discussion is to raise the hypothesis that geohistorical influences are not deterministic but instead serve as fundamental causes of disparities.

To explore the link between ancient geohistorical events and current-day factors that influence disparities, the relationship between all cancer mortality and Black Belt residence was evaluated for 82 Black Belt counties and 370 non-Black Belt counties in 5 core Black Belt states (AL, GA, MS, NC, SC) using Surveillance, Epidemiology and End Results (SEER) cancer rates (2015–2019) using the Vintage 2020 mortality files and 2020^18^ U.S. Census Small Area Income and Poverty Estimates (SAIPE). Black Belt counties had a significantly higher death rate than non-Black Belt counties with mean age-adjusted mortality in Black Belt counties of 181.8 per 100,000 versus non-Black Belt counties of 171.6 (*p*-value from Wilcoxon rank sum test <0.001).

Similarly, Black Belt counties had significantly higher poverty rate than non-Black Belt counties with mean age-adjusted percentage of resident living in poverty in Black Belt counties of 23.8% versus non-Black Belt counties of 16.5% (*p*-value from Wilcoxon rank sum test <0.001). After adjusting for percentage poverty and state, the effect of Black Belt county on all cancer mortality became nonsignificant (*p*=0.348). This suggests, as expected, that sociodemographic factors are the explanation for the high cancer rates in Black Belt counties. These data argue against geographic determinism in health disparities.

Understanding and mitigating inequities require a multisector approach that can benefit from considering intersectionality of multiple factors.^19^ Warnecke et al^1^ provided a conceptual framework for consideration of health inequities that includes factors acting at distal (i.e., social conditions and policies; institutional context), intermediate (i.e., social context, social relationships, and physical relationships) and proximal (i.e., individual demographics and risk behaviors; biological pathways and responses) levels. All of these levels have been influenced by upstream contributions of geohistorical events, which could be considered to provide a better understanding of the fundamental causes of health inequities. Even genetics and biology are known to be influenced by population genetics forces such as selection that result in the current distribution of genetic, genomic, and biological traits that contribute to health and health inequities.^20^

Already multisector teams are addressing critical public health problems in teams that include epidemiologists, behavioral scientists, basic laboratory sciences, clinical caregivers, health systems experts, and others. These disciplines are increasingly seeking the contributions of geographers, historians, political scientists, policy makers, engineers, geologists, economists, and others. The collaboration of experts from this expanded set of disciplines—and the data, methods, and perspectives they bring—are needed to address the complex intersectionality of cancer causation and cancer inequities.

## Abbreviation Used

SAIPE: Small Area Income and Poverty Estimates
